# Peroneal groove deepening as the treatment of peroneal tendon subluxation: A case report

**DOI:** 10.1016/j.ijscr.2019.11.015

**Published:** 2019-11-19

**Authors:** Ihsan Oesman, Dody Kurniawan, Rio Wikanjaya

**Affiliations:** a Department of Orthopaedic and Traumatology, Faculty of Medicine Universitas Indonesia, Dr. Cipto Mangunkusumo Hospital, Jakarta, Indonesia; b Department of Orthopaedic & Traumatology, Cipto Mangunkusumo National Central Hospital and Faculty of Medicine, Universitas Indonesia, Jalan Diponegoro No. 71, Central Jakarta, Jakarta, 10430, Indonesia

**Keywords:** Peroneal tendon subluxation, Peroneal tendon dislocation, Ankle instability, Peroneal groove deepening, Retinaculum ligament repair

## Abstract

**Introduction:**

Peroneal tendon subluxation is a relatively rare disorder that is often misdiagnosed as an ankle sprain. It affects mainly young adults, usually during sports activities. It is mostly caused by avulsion of the superior peroneal retinaculum (SPR) from its fibular insertion, which requires surgical intervention, especially for cases of symptomatic chronic peroneal tendon subluxation and/or dislocation. We reported a case of peroneal tendon subluxation of left ankle treated with peroneal groove deepening and retinaculum ligament repair, the objective of which is to illustrate the effectiveness of this procedure.

**Case presentation:**

We present a case of a thirty-four-year old male with peroneal tendon subluxation of left ankle. The patient was injured while exercising two years prior to admission. He now reported pain and instability on the left ankle. We performed peroneal groove deepening and retinaculum ligament repair to treat this patient.

**Discussion:**

Peroneal groove deepening and retinaculum ligament repair shows an excellent clinical outcome; after the procedure, there are no major complications such as infections, wound problems or permanent discomfort. Overall, the result of surgery had been considered satisfactory. Postoperatively, the ankle was placed in a below-knee, non–weight-bearing temporary cast in semi-equinus position for two weeks.

**Conclusion:**

The incidence rate of peroneal tendon subluxation is relatively low, and surgery is the primary treatment of this type of injury, with various available surgical methods available for the performing surgeon. Peroneal groove deepening and retinaculum ligament repair offers a satisfactory outcome.

## Highlights

• Subluxation of the peroneal tendon is relatively infrequent.
• Subluxation of the peroneal tendon often misdiagnosed as ankle sprain.
• Avulsion of the superior peroneal retinaculum from is the main cause of peroneal tendon subluxation.
• Good outcomes can be achieved by peroneal groove deepening and retinaculum ligament repair.

## 1 Introduction

Dislocation or subluxation of the peroneal tendon is a relatively rare occurrence and is often misdiagnosed as an ankle sprain, particularly in the acute setting. It affects mostly young adults, mainly the ones who are involved in sports activities. The dislocation itself is mostly caused by avulsion of the superior peroneal retinaculum (SPR) from its fibular insertion [1]. The peroneal muscles functions as dynamic stabilizers of the ankle and are essential in terms of proprioception, independent from its status of the lateral ankle ligaments. A poor peroneal function can give patients a sense of instability, even in a mechanically stable ankle. This may cause persistent pain and swelling after an ankle injury. Although the true incidence of peroneal tendon tear is currently unknown, the estimates shows a range of 11%–37% in cadaveric samples, and up to 30% in patients undergoing surgery for ankle instability [2]. Unlike the Achilles and the posterior tibialis tendons however, the peroneal tendons possesses a more than adequate vascularization and is almost completely unaffected by degeneration due to inadequate blood supply [3].

Peroneal tendon subluxation mainly occurs after a forceful contraction of the respective muscles when the foot is in the dorsiflexion position, with or without an inverted position of the foot and ankle. Surgical intervention remains the mainstay treatment for symptomatic chronic peroneal tendon subluxation and/or dislocation. We report a case of peroneal tendon subluxation of left ankle which is treated with peroneal groove deepening and retinaculum ligament repair. This study aims to illustrate the effectiveness of these procedures in treating peroneal tendon subluxation. This paper is written in concordance to the SCARE criteria [4].

## 2 Case presentation

We present a case of a thirty-four-year-old male with pain on his left ankle two years before admission. The pain was first felt two years ago when the patient got injured while playing futsal. It subsided after a few weeks. The injury leaves an odd sensation on the ankle as felt by the patient whenever he is sitting or kneeling; he could feel his tendon ankle moving to anterior part of his ankle and the patient could reverse this by moving the tendon manually to the posterior. After several months the symptoms worsens and started to affect the patient’s mobility and activities. The patient then came to orthopaedic surgeon in Jayapura and was referred to Cipto Mangukusumo National Hospital. There was neither history of any comorbidities nor previous surgery on this patient.

**Fig. 1:**
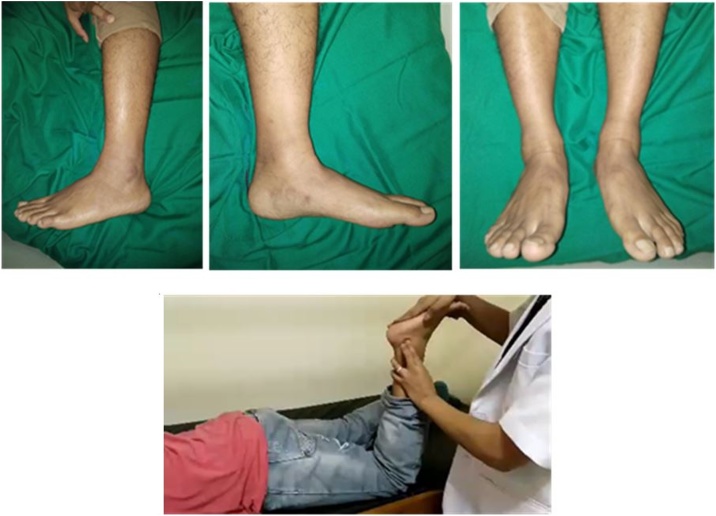
a. Pre-operative clinical examination showing no deformity or wound; b. Peroneal longus tendon dislocation test.

Local physical examination of the left ankle reveals no deformity or wound (Fig. 1a). The pain was felt at lateral side of ankle VAS 2–3 on palpation. The distal sensory was normal, as well as the range of movement, including dorsiflexion and eversion-inversion of ankle. The peroneal longus tendon dislocation test was positive (Fig. 1b).

**Fig. 2:**
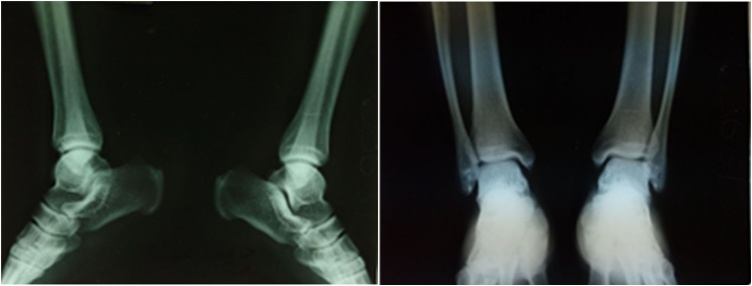
Pre-operative X-ray of both ankle.

**Fig. 3:**
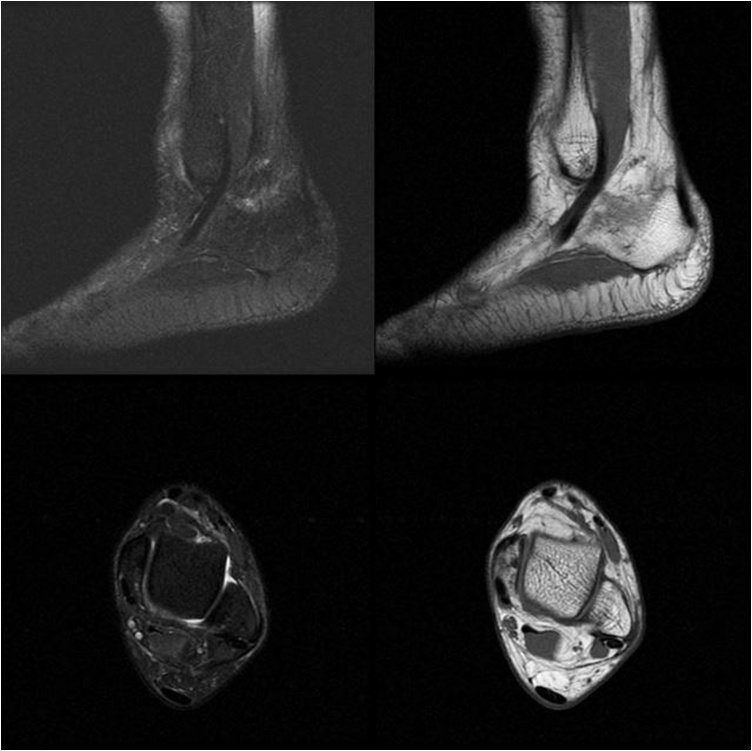
MRI showing peroneal tendon subluxation.

The anteroposterior X-ray of the ankle we obtained was normal (Fig. 2), while MRI examination showed a tendon displacement (Fig. 3).

**Fig. 4:**
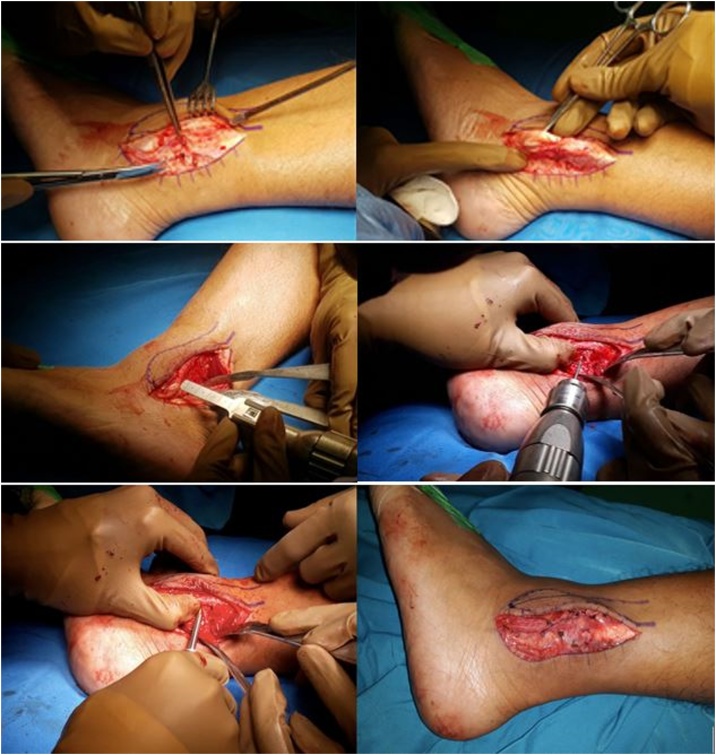
a. Dissection at retinaculum ligament and resection of fibrotic tissue at peroneus longus tendon; b. Osteotomy at the tip of lateral malleolus and drilling of intramedullary; c. Fixation of the posterolateral and posteromedial edges of peroneal groove with cortical screw and repair of retinaculum ligament.

Based on clinical and radiological examination, we established the diagnosis of the Peroneal tendon subluxation of left ankle. We performed Peroneal groove deepening and Retinaculum ligament repair procedure to treat this condition (Fig. 4).

The patient was put in a supine position with a bolster under the ipsilateral hip in order to facilitate internal rotation and expose the respective extremity. A 6-cm to 8-cm longitudinal incision was made over the posterolateral aspect of the distal fibula, extending to the fibular malleolus, after which anatomical dissection and hemostasis were conducted gradually to expose the deep fascia and peroneal retinaculum. This was followed by debridement of the damaged tendon and resection of non-viable fibrotic tissue and osteotomy at 5 cm proximal to the tip of the lateral malleolus.

Multiple drill holes were made along the posterolateral and posteromedial edges of the groove for approximately 3 cm, which were then connected with a sharp osteotome. Once the peroneal tendons were reduced, the posterolateral surface of the fibula was roughened using a nibbler or osteotome so that the repaired retinaculum will be scarred down onto it. The Superior Peroneal Retinaculum (SPR) was then re-attached with an absorbable suture and fixation performed with cortical screw 1.7. The wound was then irrigated and closed in layers.

Postoperatively, the ankle was placed in a below-knee, non–weight-bearing temporary cast, in the semi-equinus position for 2 weeks. The cast was then changed to a walker boot (fully weight-bearing) for 4 weeks after which the patient could start passive physiotherapy. At the 6-week stage, the patient could fully mobilize while weight-bearing and a full active physiotherapy program was started.

## 3 Discussion

Peroneal tendon subluxation and dislocation invariably occurs to secondary disruption or elevation of the superior peroneal retinaculum (SPR) from its insertion along the posterolateral margin of the fibula, with or without displacement of the associated fibro-cartilaginous ridge or cortical fibular fragment.

Anatomically, the common peroneal sheath passes through a fibro-osseous tunnel known as the retro-malleolar groove. This groove is covered with fibrous cartilage with the peroneus longus tendon lying posterolateral to the peroneus brevis tendon. The groove itself varies in depth and shape, a determinant of which is a cartilaginous ridge, which enhances the depth of the groove. The SPR is a fibrous band that originates from the posterolateral aspect of the fibula, passes over the tendons and inserts most commonly onto the calcaneus and Achilles tendon sheath, although there are several distinct insertional variations [1].

The typical cause of this type of injury is the sudden dorsiflexion stress caused by a violent reflex contraction of the peroneal musculature, which is also true for any dislocation or subluxation that occurs during sporting activities. This particular condition includes the sudden, reflexive contraction of the peroneal muscle during dorsiflexion of the everted foot or an acute inversion injury to the dorsiflexed ankle [5]. The patient exhibiting a chronic dislocation of the peroneal tendons poses a more complicated problem when diagnosing it; the patient would sometimes exhibit repeated episodes of complete dislocation of the tendons and can even demonstrate this for the clinician. A typical patient will report a history of a feeling of instability, occasional subluxation or possibly a clicking feeling behind the lateral malleolus [6].

Anatomic factors such as the shape and texture of the fibular groove have been described as a possible factor that contributes to subluxation of the peroneal tendons; the dense fibrocartilaginous ridge on the posterior aspect of the fibula effectively adds to the overall depth of the groove and in some instances, it may be small or even nonexistent, affecting the stability of the tendons behind the fibula. An acute event such as the dorsiflexion of the ankle with the foot inverted or everted, can more easily disrupt the attachment of the superior peroneal retinaculum, which can compromise the function of the retinaculum and result in peroneal tendon subluxation. The contraction of these tendons within a shallow peroneal groove can also result in dislocation [7].

**Fig. 5:**
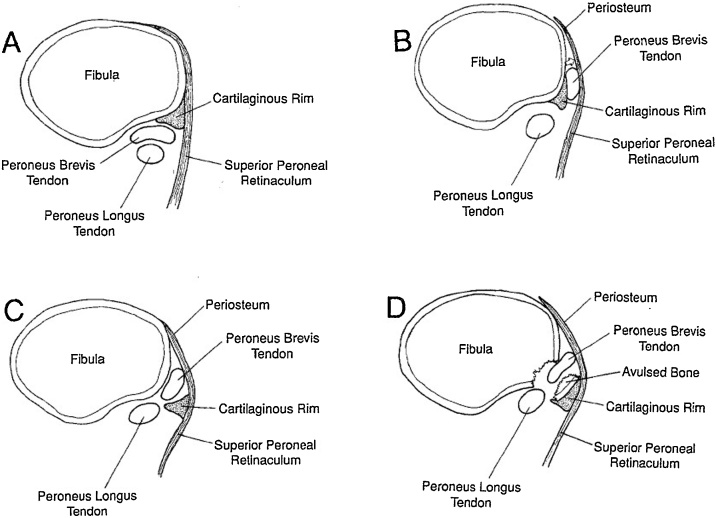
Classification of pathology in peroneal tendon dislocations. (A) Normal. (B) Grade I (C) Grade II (D) Grade III.

Eckert and Davi classified the acute injury pattern into the following three types (Fig. 5) [6]:

Grade I: The retinaculum, which is merged with the periosteum on the lateral aspect of the fibula, is stripped away from the posterolateral aspect of the distal end of the fibula, resulting in dislocation of the tendons into this pouch. As a result, the tendons lie between the periosteum and the fibula.

Grade II: The cartilaginous ridge along the lateral aspect of the fibula is avulsed with the periosteum over the fibula and the tendon subluxated into this pouch.

Grade III: The least common type, a bony avulsion of the posterolateral aspect of the fibula containing the cartilaginous rim and a flake of bone permitting the tendon to slide beneath the periosteum.

The diagnosis of peroneal tendon subluxation or dislocation was made carefully by history taking, physical examination, and radiograph imaging. In chronic peroneal tendon subluxation or dislocation there would often be a history of popping or snapping tendons, as well as an easily visible dislocated tendon lying on top of the fibula. In our case, the patient was able to repeatedly demonstrate the tendons dislocating. There was also a history of instability of the ankle. In the chronic setting, there is little tenderness or swelling, and the ligaments are usually intact, with a stable anterior drawer test. The tendons may freely dislocate on activation of the peroneals with dorsiflexion and eversion, and the patient may be able to demonstrate this. If the dislocation is not apparent, a guiding digit can help find it. The use of a guiding digit is often more accessible with the patient on his or her side and fully relaxed on an examination couch [8].

An everyday radiographic examination may reveal the small avulsion flake on an internally rotated oblique view, however, the radiographs often appears normal. A computed tomography scan allows for a more precise definition of the retromalleolar sulcus and the position of the peroneal tendons, while magnetic resonance imaging wold be able to define the soft tissue structures such as the pouch between the periosteum and the fibula, which, on some occasions, will contain the peroneal tendons [6].

Peroneal tendon dislocation treatment should be based on whether it is an acute or chronic injury and whether the patient is an athlete. Non-athletes with an acute dislocation may be offered conservative management but should be warned beforehand that there is a 50% chance of recurring dislocation. In case of unsuccessful conservative management or chronic instability, surgical intervention is advised [9]. There are various surgical techniques to treat peroneal dislocation, however, no randomized studies have been conducted to determine which method of treatment is superior, and the available literature is limited to case reports and small case series. There are five categories of surgical repair: (1) anatomic reattachment of the retinaculum, (2) reinforcement of the superior peroneal retinaculum with local tissue transfers, (3) rerouting the tendons behind the calcaneofibular ligament, (4) bone block procedures, and (5) groove deepening procedures. A systematic review describing the surgical treatment of peroneal tendon dislocation reported that most procedures included in the reviewed studies resulted in right to excellent outcomes, low recurrence rates, and a high rate of return to sports [10]. Mu Hu and Xiangyang Xu investigated the clinical efficacy of a modified approach for the treatment of chronic subluxation of the peroneal tendons using posteromedial peroneal tendon groove deepening combined with repair of the tendon sheath, which resulted in satisfactory efficacy for the treatment of chronic subluxation of the peroneal tendons [11]. Ziai et al. reported that combined treatment of peroneal tendon dislocation and coexisting lateral and medial ligamentous laxity in the ankle joint following arthroscopy results in excellent clinical outcomes and high patient satisfaction [12]. There have been reports of significant complications following open surgical procedures (infection, wound problems, bone block fracture, permanent discomfort, recurrent subluxation, and skin hypersensitivity), however, overall outcome of the various surgery have been considered good, with a high rate of return to actively doing sport activities [13].

Post-operatively, the foot is placed in a non-weight bearing short-leg cast in a slightly inverted position for the first two weeks. After two weeks, a short-leg walking cast in neutral position is was applied for four more weeks, allowing weight-bearing as tolerated by the patient. Running and jogging with brace are allowed at 8–10 weeks. At 8–10 weeks, peroneal strengthening is initiated. After completion of a progressive strengthening exercise program of the extrinsic foot muscles, cutting activities and an expected return to sports are allowed at 4–6 months [14].

## 4 Conclusion

The peroneal tendon subluxation incidence is relatively low and it is often mistaken for a sprain of the lateral malleolus. This injury may significantly degrade the function of the ankle when it becomes chronic; thus a timely diagnosis and effective therapy is of utmost importance. Symptomatic chronic peroneal tendon dislocation or subluxation is easier to diagnose and requires operative management. Although there is a general agreement on the effectiveness of the surgical approach, no randomized control trials are available to determine relative effectiveness of one surgical technique over another. Nevertheless, groove deepening procedure, including special attention to maintain a smooth gliding surface for the tendons, as well as a combination with retinacular repair and re-attachment appears as a logical, anatomic, and reproducible technique that provides adequate stability, function, and excellent outcomes.

## Funding

The authors received no financial support for the research, authorship, and/or publication of this article.

## Ethical approval

The ethical approval was not required for this case report.

## Consent

Informed consent had been obtained from the patient before the manuscript was written.

## Author contribution

Ihsan Oesman: Concept of the study, data collection & interpretation, and writing the paper.

Dody Kurniawan: Data collection, data interpretation and writing the paper.

Rio Wikanjaya: Data collection, data interpretation and writing the paper.

## Registration of research studies

Case report needs no research registration.

## Guarantor

Ihsan Oesman.

## Provenance and peer review

Not commissioned, externally peer-reviewed.

## Declaration of Competing Interest

The authors declare that there is no conflict of interest regarding the publication of this article.
